# Reply to Letter to the Editor

**DOI:** 10.3906/sag-2002-224

**Published:** 2020-06-23

**Authors:** Zehra İpek ARSLAN

**Affiliations:** 1 Department of Anesthesiology and Reanimation, Faculty of Medicine, Kocaeli University, Kocaeli Turkey

**To the Editor, **

On behalf of my coauthor, I would like to thank in advance the authors, who have been referenced in our published trial, for their analyses and consideration in their comparisons of nostril selection, the need for the use of certain manoeuvres to direct the tube into the trachea during nasotracheal intubation with the Airtraq NT® [1].

Takasugi et al. [2] applied nasotracheal intubation first through the right nostril without any view, using any manoeuvre necessary in order to improve the view or to direct the tube into the trachea. Without these aids, intubation was successful in 85% of patients when they initially inserted the tube form the right nostril. We do not know what the results would have been if they had used the left nostril first. Moreover, this example was not a clinical study, as this fact was mentioned as a limitation in their study.

We asked our patients in our daily practice from which nostril airflow was easier, this question was also asked in the Smith and Reid published trial [3]. Then we tried to insert the tube and if we felt any resistance then we tried the opposite nostril. I strongly agree with the authors that we must prepare both nostrils. In the event of difficulty then an attempt should be made through the other nostril and a collaborative decision also be made considering the surgeon’s preferences in order to improve teamwork. 

If authors look again at the results section of our study, they will see that the insertion times were comparable among the groups in Table 2 (P = 0.293). The intubation and total intubation times were statistically significantly shorter in the right nostril group (P < 0.001). 

I am grateful to our colleagues for recognizing and paying attention to the difference between the Sellick manoeuvre (cricoid pressure) and the external laryngeal manipulation (BURP) manoeuvre to improve our knowledge further as practitioners. Fundamentally, we use all necessary manoeuvres to improve the view depending on the patients’ condition and subsequent response to the procedure. 

In the discussion section, we note our recommendation for future trials, providing the aforementioned references. However, the references cited do not focus on nasotracheal intubation; rather they are related to certain manoeuvres that can be applied to aid in successful nasal fiber-optic intubation or orotracheal intubation with the Airtraq NT® for use in directing the tube into the trachea (90° anticlockwise rotation, cricoid pressure, and head extension). Moreover, tube selection, such as the Parker –Flex-Tip tube, facilitated tube advancement through the vocal cords and decreased the occurrence of traumatic complications during fiber-optic intubation. Additionally, as an alternative, these could be used with the Airtraq NT® or other videolaryngoscopes for nasotracheal intubation in future trials [4–6]. Furthermore, production of a left sided Airtraq is something we hope for in the future. 

Kind regards, 

**Key words:** Airtraq, videolaryngoscope, nasotracheal, right, nostril, 90° anticlockwise, intubation

## Conflict of interest

The authors have no financial or competing interest.
